# Supplementary material for: Systematically uncovering the absorbed effective substances of Radix Scutellaria-licorice drug pair in rat plasma against COVID-19 using a combined UHPLC-Q-TOF-MS analysis and target network pharmacology
Source: PLoS One. 2023 Aug 9;18(8):e0289121. doi: 10.1371/journal.pone.0289121 (PMC10411761; doi:10.1371/journal.pone.0289121)
Supplement: S1 File — (DOCX) [file pone.0289121.s001.docx]

Table S1 List of intersections targets and involved-compounds

| **Compound** | **Target** |
| --- | --- |
| MOL000392 | IL2, ALDH2, ABCG2, EGFR, ABCB1, ESR1, TLR9, CA2, DHODH, FEN1, PTPN1, |
| MOL000008 | ESR1, PTGS2, GSK3B, ABCC1, TTR, CSNK2A1, ABCG2, ALOX5, PARP1, CA2, ABCB1, APP, MMP12, AHR, AR, EGFR, CDK1, LCK, AXL, CDK2, F2, PLG, PIK3R1, MMP3, AKT1, |
| MOL000525 | CDK1, IKBKB, ABCB1, CA2, ABCG2, PTGS2, NOS2, ESR1, AR, GSK3B, ABCC1, TTR, CSNK2A1, EGFR, ALOX5, LCK, AHR, PIK3CG, PARP1, APP, AXL, KIT, MMP12, F2, CDK2, PTPN1, PIK3R1, |
| MOL100000 | EGFR, ABCG2, APP, F3, NOS2, SNCA, ALDH2, ALOX5, PTGS2, ABCB1, ESR1, TLR9, CTSL, PTPN1, ABCC1, CSNK2A1, DHODH, AHR, CDK1, MMP12, CDK2, |
| MOL004953 | MMP12, ABCC1, SHBG, |
| MOL100014 | PTGS2, NOS2, KIT, AR, ABCB1, ABCG2, IKBKB, |
| MOL004915 | PTPN1, ESR1, ALDH2, ABCB1, ABCG2, AR, HSP90AA1, EGFR, IL2, ADRB1, CHEK2, BCL2L1, RELA, |
| MOL100016 | PTGS2, NOS2 |
| MOL100011 | AR, ESR1, CA2, ABCB1, ABCG2, APP, GSK3B, ABCC1, CDK1, CSNK2A1, TTR, ALOX5, PTGS2, |
| MOL001801 | CA2, ERN1, HDAC6, DPP4, ALB, MCL1, |
| MOL004884 | AR, PTPN1, SIGMAR1, SHBG, NOS2, ALOX5, MAPK3, IDO1, CES1, NR3C2, PPARG, ESR1, TNF, G6PD, CYP51A1, CD81, IL6, PTGS2, MMP3, MMP1, |
| MOL009290 | TNF, IL2, ALDH2, HSP90AA1, CA2, |
| MOL100005 | CA2, TTR, COMT, BCL2L1, SERPINE1, ALB, |
| MOL100008 | NOS2, PTGS2, ABCB1, KIT, MCL1, PLG, ABCG2, PIK3CG, HSP90B1, APP, AHR, IKBKB, HSP90AA1, F2, PIK3R1, MMP3, AKR1A1, GSK3B, CDK1, EGFR, CA2, LCK, ALOX5, TTR, AR, PDE5A, MMP12, ABCC1, FEN1, |
| MOL004835 | PTPN1, F3, ABCG2, APP, PTGS2, ALOX5, EGFR, NOS2, ABCB1, CDK1, CAPN1, ESR1, BCL2L1, BCL2, MMP1, ALDH2, ADAM17, MMP3, ABCC1, SHBG, TLR9, PPARG, VCP, |
| MOL100020 | ABCG2, ERN1, PTGS2, DHFR, AR, MMP1, CDK1, ALOX5, GSK3A, CASP4, CASP6, HDAC6, PARP1, NOS3, HMOX1, TTR, CA2, CES1, JAK1, |
| MOL004896 | BCL2L1, JUN, AR, ADRA1A, CYP2D6, VDR, IL2, STAT3, |
| MOL002927 | KIT, ABCG2, PTGS2, ABCB1, MCL1, PLG, ALOX5, NOS2, APP, ABCC1, CA2, AHR, MMP3, PLA2G7, EGFR, PIK3CG, GSK3B, MMP1, ADAM17, ERN1, PPIA, IDO1, CSNK2A1, CDK1, SLC29A1, |
| MOL002936 | KIT, PTGS2, NOS2, PLG, ABCG2, AHR, ABCB1, TTR, AR, PIK3R1, AXL, AKR1A1, CDK1, CA2, GSK3B, IKBKB, ALOX5, PIK3CG, SIGMAR1, EGFR, ABCC1, MCL1, |
| MOL004894 | BCL2L1, JUN, AR, STAT3, MMP12, ADRA1A, CYP2D6, IL2, PTPN1, |
| MOL002560 | CA2, ABCB1, ABCG2, ESR1, PARP1, CDK1, CSNK2A1, PTGS2, GSK3B, ABCC1, TTR, LCK, IKBKB, ALOX5, EGFR, AR, APP, MMP12, AHR, PRKDC, PIK3CG, KIT, PTPN1, NOS2, PLA2G4A, AXL, PDE5A, PLG, F2, SIGMAR1, |
| MOL004568 | MMP12, ABCB1, CA2, APP, MAPK14, STAT1, |
| MOL100019 | APP, ABCG2, F3, ALDH2, |
| MOL100003 | CA2, TNF, PTGS2, IL2, |
| MOL000390 | ALDH2, ESR1, EGFR, ABCG2, IL2, ABCB1, CA2, TLR9, ALOX5, PTPN1, PLAT, F10, MCL1, PPARG, |
| MOL100017 | PTGS2, TNF, IL2, CA2, ALOX5, |
| MOL004926 | ALDH2, MMP12, ABCC1, SHBG, ABCG2, ESR1, MMP1, SLC29A1, |
| MOL013068 | IL2, CA2, TNF, PTGS2, MAPK14, |
| MOL002910 | ESR1, ABCC1, SHBG, ABCG2, MMP12, CES1, PPARG, MAPK14, BCL2, APP, CA2, AKT1, ABCB1, KIT, GRM2, SERPINE1, SNCA, DNMT1, STAT1, CTSB, |
| MOL100002 | TNF, IL2, EGFR, CA2, ALDH2, PTGS2, |
| MOL004328 | ABCC1, SHBG, ESR1, ABCG2, CES1, PPARG, MMP12, SERPINE1, CA2, KIT, CTSB, F3, BCL2L1, PIK3CB, CYP3A4, PIK3CA, VCP, VEGFA, ERN1, MMP3, APP, GSK3B, BCL2, LCK, CDK4, SNCA, |
| MOL001789 | EGFR, ABCG2, APP, F3, NOS2, SNCA, ALDH2, ALOX5, PTGS2, ABCB1, ESR1, TLR9, CTSL, PTPN1, ABCC1, CSNK2A1, DHODH, AHR, MMP12, CDK1, CDK2, |
| MOL004892 | BCL2L1, AR, STAT3, F2, VDR, GRB2, ADRA1A, CYP2D6, IL2, |
| MOL004564 | ABCB1, ABCC1, CA2, MCL1, ABCG2, ALOX5, AHR, EGFR, PIK3CG, F2, CDK1, PIK3R1, GSK3B, MMP3, CSNK2A1, AKT1, AXL, AKR1A1, CDK2, KIT, PLG, APP, ESR1, PTPN1, |
| MOL100010 | TNF, IL2, CA2, PTGS2, |
| MOL002933 | NOS2, PTGS2, MCL1, ESR1, EGFR, APP, ABCG2, KIT, ALOX5, ABCB1, ABCC1, CDK1, CA2, AHR, PIK3CG, IKBKB, GSK3B, TTR, CSNK2A1, AR, F2, PARP1, MMP12, PLG, PIK3R1, MMP3, |
| MOL002919 | CDK1, ABCB1, CA2, ABCG2, ESR1, PTGS2, IKBKB, AR, GSK3B, ABCC1, TTR, CSNK2A1, LCK, PARP1, ALOX5, APP, NOS2, MMP12, EGFR, AHR, KIT, PIK3CG, AXL, PTPN1, PRKDC, MAPK3, SIRT1, PLA2G4A, CDK2, |
| MOL100001 | BCL2L1, JUN, AR, F2, STAT3, VDR, ADRA1A, CYP2D6, IL2, |
| MOL100006 | TNF, IL2, CA2, SLC29A1, EGFR, MMP1, PTGS2, LGALS3, LGALS9, |
| MOL100004 | TNF, IL2, MAPK14, PTGS2, IMPDH2, |
| MOL100015 | IKBKB, F10, ABCB1, CDK1, AHR, TTR, AR, GSK3B, AKT1, AXL, AKR1A1, |
| MOL004876 | BCL2L1, JUN, AR, STAT3, MMP12, ADRA1A, CYP2D6, IL2, PTPN1, |
| MOL000103 | CA2, HDAC6, ERN1, |
| MOL003044 | ABCC1, CA2, PLG, APP, ALOX5, PARP1, ABCB1, ABCG2, GSK3B, TTR, MMP12, ESR1, CSNK2A1, EGFR, CDK2, PTGS2, MCL1, F2, CDK1, PIK3R1, MMP3, AKT1, AXL, AKR1A1, |
| MOL000173 | PTGS2, NOS2, KIT, ABCB1, IKBKB, CDK1, ABCG2, ESR1, EGFR, CA2, MCL1, PIK3CG, AR, LCK, MMP12, PIK3R1, MMP3, AKR1A1, HSP90AA1, ALOX5, APP, HSP90B1, ABCC1, TTR, PDE5A, GSK3B, |
| MOL012245 | ABCG2, SHBG, ESR1, ABCC1, MMP12, CES1, CA2, GRM2, DNMT1, STAT1, PLG, KIT, APP, MAPK14, |
| MOL002935 | TNF, IL2, EGFR, ALDH2, |
| MOL004804 | AR, PTPN1, SIGMAR1, SHBG, NOS2, ALOX5, MAPK3, IDO1, CES1, NR3C2, PPARG, ESR1, TNF, G6PD, CYP51A1, CD81, IL6, PTGS2, MMP3, |
| MOL100018 | IKBKB, AR, CDK1, ABCB1, PTGS2, ABCG2, AHR, KIT, GSK3B, CSNK2A1, PIK3R1, MMP3, AKR1A1, PARP1, MMP12, COMT, CDK2, |
| MOL000417 | ABCB1, ESR1, TLR9, EGFR, ALDH2, ABCG2, CA2, IL2, PTPN1, ABCC1, PLAT, F10, |
| MOL002714 | CDK1, ABCB1, CA2, ABCG2, ESR1, PTGS2, IKBKB, AR, GSK3B, ABCC1, TTR, CSNK2A1, LCK, PARP1, ALOX5, APP, NOS2, MMP12, EGFR, AHR, KIT, PIK3CG, AXL, PTPN1, PRKDC, MAPK3, SIRT1, PLA2G4A, CDK2, |
| MOL003837 | EGFR, CA2, CCND1, CDK2, GSK3B, CSNK2A1, AKT1, HSPA1A, ESR1, ALDH2, SNCA, |
| MOL100012 | F10, IKBKB, ABCB1, COMT, ABCG2, ALOX5, ABCC1, AHR, ESR1, GSK3B, CSNK2A1, AR, |
| MOL004951 | SLC29A1, PTPN1 |
| MOL000105 | CA2, TTR, COMT, BCL2L1, SERPINE1, ALB, |
| MOL100013 | ABCG2, F3, ALDH2, EGFR, |
| MOL100009 | ABCB1, ESR1, CSNK2A1, ABCG2, CA2, PTGS2, GSK3B, ABCC1, TTR, CDK1, ALOX5, AXL, PARP1, AR, AHR, EGFR, LCK, APP, MMP12, IKBKB, CDK2, PTPN1, F2, PIK3CG, MCL1, PIK3R1, |
| MOL100007 | TNF, IL2 |
| MOL004883 | PTPN1, ESR1, ALDH2, AR, ABCG2, ABCB1, EGFR, HSP90AA1, BCL2L1, RELA, IL2, ADRB1, CHEK2, |
| MOL000486 | ALDH2, ESR1, EGFR, IL2, ABCB1, ABCG2, CA2, TLR9, ALOX5, |
| MOL004903 | MMP12, ABCC1, SHBG, ABCG2, MMP1, ESR1, SLC29A1, |
