# Supplementary material for: Systematically uncovering the absorbed effective substances of Radix Scutellaria-licorice drug pair in rat plasma against COVID-19 using a combined UHPLC-Q-TOF-MS analysis and target network pharmacology
Source: PLoS One. 2023 Aug 9;18(8):e0289121. doi: 10.1371/journal.pone.0289121 (PMC10411761; doi:10.1371/journal.pone.0289121)
Supplement: Attachment — Submitted filename: Response to Reviewers.docx [file pone.0289121.s002.docx]

Dear Editor,

Thank you for your letter and comments concerning our manuscript entitled '' Systematically uncovering the absorbed effective substances of Radix Scutellaria-licorice drug pair in rat plasma against COVID-19 using a combined UHPLC-Q-TOF-MS analysis and target network pharmacology''. (Manuscript Number: PONE-D-23-05859). We have carefully revised the manuscript in accordance with the reviewers’ comments and read the manuscript to minimize typographical, grammatical, and bibliographical errors. The revised portion is marked in red bold. We would like to present the alterations and explanations to your comments point-by-point as follows:

**Reviewer #1:**

**Comment 1:** The English is too poor.

**Reply:** Thanks for your comments. We have tried our best to edit the language in the revised manuscript.

**Comment 2:** In section 2.7.1, Please describe how to search targets by Swiss Target Prediction, using a SMILES or draw a molecule.

**Reply:** Thanks for your comments. We have added more details on how to search for targets from the Swiss Target Prediction database in section 2.7.1.

**Comment 3:** The network pharmacology should add the Gene Ontology and KEGG analysis.

**Reply:** Thank you very much for your helpful suggestion. Based on your suggestions, the Gene Ontology and KEGG analysis have been added to the revised manuscript.

**Reviewer #2:**

**Comment 1:**  The text contains grammatical errors throughout the manuscript. Punctuation and spacing are often missing, making it difficult to read. The reference formatting appears to be inconsistent. A thorough proofreading and editing are necessary.
• e.g. In abstract: "Besides, we optimized the conventional process ways of network pharmacology...", consider rephrasing it to "Additionally, we optimized the conventional methodologies of network pharmacology..." for better clarity.
• “A total of 85 absorbed constituents were identified or tentatively characterized in dosed plasma, including 32 components in Radix Scutellaria, 27 components in Licorice and 65 components in RSLDP.” There is lack of space after each punctuation, and the sentence is unclear and hard to follow.

**Reply:** Thank you very much for your helpful suggestion. We were really sorry for our careless mistakes. We have revised the grammar, spelling and reference formatting of the entire manuscript.

"Besides, we optimized the conventional process ways of network pharmacology..." have changed to "Additionally, we optimized the conventional methodologies of network pharmacology...".

“A total of 85 absorbed constituents were identified or tentatively characterized in dosed plasma, including 32 components in Radix Scutellaria, 27 components in Licorice and 65 components in RSLDP” have changed “A total of 85 absorbed constituents were identified or tentatively characterized in dosed plasma, including 32 components in the group of Radix Scutellaria, 27 components in the group of Licorice, and 65 components in the group of RSLDP”.

**Comment 2:** There is a lack of context and background information in the results and discussion section. It would be helpful for the authors to better explain the rationale behind the study, the significance of the findings, and how they relate to existing knowledge in the field.

**Reply:** We sincerely appreciate the valuable comments. We have added more context and background information in the results and discussion section of the revised manuscript.

**Comment 3:** The manuscript would benefit from a more detailed discussion of the limitations of the study, as well as suggestions for future research. Section 3, which is titled “results and discussion”, does not adequately discuss the gap between the results and the literature. Only four pieces of literature are cited in this section, which is unusual for a discussion section.

**Reply:** Thanks a lot for your meaningful comments**.** We have fully described the limitations and prospects of the discussion section. And we have adequately discussed the relationship between the experimental results and the references in the Results and Discussion section of Section 3. We have added more references to support our idea in the revised manuscript.

**Comment 4:** The figures should be clear and of high quality.
e.g. Figure 9 has been stretched strangely. Some gene names are not fully displayed or difficult to read.

**Reply:** Thank you very much for your comments. The quality and clarity of Figure 9 has been improved.

**Comment 5:** The format of references is seriously inconsistent. The references should be formatted consistently, and the authors should ensure that all cited literature is relevant and up-to-date.

**Reply:** Thanks a lot for your comments. We are sorry for our carelessness. Based on your comments, we have made the corrections to make the formatting of references consistent. And we have ensured that all literature cited is relevant and up-to-date.
