# Supplementary material for: Systematically uncovering the absorbed effective substances of Radix Scutellaria-licorice drug pair in rat plasma against COVID-19 using a combined UHPLC-Q-TOF-MS analysis and target network pharmacology
Source: PLoS One. 2023 Aug 9;18(8):e0289121. doi: 10.1371/journal.pone.0289121 (PMC10411761; doi:10.1371/journal.pone.0289121)
Supplement: Attachment — Submitted filename: Response to Reviewers.docx [file pone.0289121.s003.docx]

Dear Editor,

Thank you for your letter and comments concerning our manuscript entitled '' Systematically uncovering the absorbed effective substances of Radix Scutellaria-licorice drug pair in rat plasma against COVID-19 using a combined UHPLC-Q-TOF-MS analysis and target network pharmacology''. (Manuscript Number: PONE-D-23-05859R2). We have carefully revised the manuscript in accordance with the editors’ comments and read the manuscript to minimize typographical, grammatical, and bibliographical errors. The revised portion is marked in red bold. We would like to present the alterations and explanations to your comments point-by-point as follows:

**Editor #1:**

**Comment 1:** The acknowledgment section does not require thanking the reviewer; Thank you to the laboratory personnel and suggest specific names; Co authors should not be reflected in the acknowledgment section. Suggest adding information such as funding to support this research.

**Reply:** Thanks for your comments. We have revised the acknowledgment section of the revised manuscript. We have added funding sources to the funding information section of the submission system.

**Editor #2:**

**Comment 1:**  We therefore request that you revise the text to fix the grammatical errors and improve the overall readability of the text. We suggest you have a fluent English-language speaker thoroughly copyedit your manuscript for language usage, spelling, and grammar. If you do not know anyone who can do this, you may wish to consider employing a professional scientific editing service.

**Reply:** Thank you very much for your helpful suggestion. We have tried our best to revise the grammar, spelling, and language usage of the entire manuscript.

**Journal Requirements #3:**

**Comment 1:**  Please review your reference list to ensure that it is complete and correct.

**Reply:** Thanks a lot for your meaningful comments**.** We have ensured that all literature cited is complete and correct.

.
